# Peptides vs. Polymers: Searching for the Most Efficient Delivery System for Mitochondrial Gene Therapy

**DOI:** 10.3390/pharmaceutics14040757

**Published:** 2022-03-31

**Authors:** Rúben Faria, Milan Paul, Swati Biswas, Eric Vivès, Prisca Boisguérin, Ângela Sousa, Diana Costa

**Affiliations:** 1 CICS-UBI—Health Sciences Research Centre, Universidade da Beira Interior, Avenida Infante D. Henrique, 6200-506 Covilha, Portugal; ruben.faria@ubi.pt (R.F.); angela@fcsaude.ubi.pt (Â.S.); 2 Nanomedicine Research Laboratory, Department of Pharmacy, Birla Institute of Technology and Science-Pilani, Hyderabad Campus, Jawahar Nagar, Medchal, Hyderabad 500078, India; milan.paul95@gmail.com (M.P.); swati.biswas@hyderabad.bits-pilani.ac.in (S.B.); 3 PhyMedExp, Université de Montpellier, INSERM, CNRS, 34295 Montpellier, France; eric.vives@umontpellier.fr (E.V.); prisca.boisguerin@inserm.fr (P.B.)

**Keywords:** cell-penetrating peptides, mitochondrial gene therapy, mitochondrial DNA diseases, mitochondria targeting, nanodelivery systems, PEI-based complexes

## Abstract

Together with the nucleus, the mitochondrion has its own genome. Mutations in mitochondrial DNA are responsible for a variety of disorders, including neurodegenerative diseases and cancer. Current therapeutic approaches are not effective. In this sense, mitochondrial gene therapy emerges as a valuable and promising therapeutic tool. To accomplish this goal, the design/development of a mitochondrial-specific gene delivery system is imperative. In this work, we explored the ability of novel polymer- and peptide-based systems for mitochondrial targeting, gene delivery, and protein expression, performing a comparison between them to reveal the most adequate system for mitochondrial gene therapy. Therefore, we synthesized a novel mitochondria-targeting polymer (polyethylenimine–dequalinium) to load and complex a mitochondrial-gene-based plasmid. The polymeric complexes exhibited physicochemical properties and cytotoxic profiles dependent on the nitrogen-to-phosphate-group ratio (N/P). A fluorescence confocal microscopy study revealed the mitochondrial targeting specificity of polymeric complexes. Moreover, transfection mediated by polymer and peptide delivery systems led to gene expression in mitochondria. Additionally, the mitochondrial protein was produced. A comparative study between polymeric and peptide/plasmid DNA complexes showed the great capacity of peptides to complex pDNA at lower N/P ratios, forming smaller particles bearing a positive charge, with repercussions on their capacity for cellular transfection, mitochondria targeting and, ultimately, gene delivery and protein expression. This report is a significant contribution to the implementation of mitochondrial gene therapy, instigating further research on the development of peptide-based delivery systems towards clinical translation.

## 1. Introduction

Mitochondria play a key role in maintaining the normal functioning of cells, especially metabolic functions. This cell organelle has its own genome, called mitochondrial DNA (mtDNA). Mitochondrial DNA is a circular, double-stranded molecule with a size of around 16 kbp, and contains 37 genes [1]. These genes encode 13 polypeptides that participate in the oxidative phosphorylation chain, 2 rRNAs, and 22 tRNAs—all exclusive to the mitochondria [2,3]. In addition to being responsible for the production of ATP through oxidative phosphorylation, in recent years many other cellular processes have been discovered revealing the involvement of mitochondria [4,5]. Their participation in cellular mechanisms ranging from inflammation to regulation of stem cell generation [6,7], cell signaling, ion homeostasis, and metabolism of amino acids, lipids, cholesterol, steroids, and nucleotides has been demonstrated [8,9]. They also contribute to cell cycle control, cell growth, and apoptosis mechanisms [10]. In this way, mutations in mtDNA cause excessive cell death and promote the appearance of several pathologies, including metabolic and neurodegenerative syndromes linked to Parkinson’s, Alzheimer’s, amyotrophic lateral sclerosis, Huntington’s disease, and diabetes [11]. Loss of influence on cell cycle control and cell death regulation, due to mutations in mtDNA, leads to cancer and autoimmune diseases [12,13]. Currently, treatments available for diseases associated with mtDNA mutations are not effective and only serve to mitigate symptoms, without providing a cure [14,15].

In line with this, the need to develop new, effective therapies arises. Since most mitochondrial diseases are associated with mutations in mtDNA, mitochondrial gene therapy emerges as a very promising approach to treat the problem at its root [16,17,18]. This type of therapy is focused on the delivery of genetic material to the mitochondria to suppress, alter, or complement the effect of defective genes [16]. To deliver genes of interest directly into the mitochondria, it is necessary to develop a vector that allows the encapsulation of DNA while providing targeting specificity [19]. This vehicle must also protect, transport, and direct the genetic content to the mitochondria, promoting its efficient release and, thus, guaranteeing the expression of both mitochondrial genes and respective proteins [20]. Viral vectors, such as retroviruses, adenoviruses, and lentiviruses, have been widely used in gene delivery due to their ability to transfect cells [21]. Among these vectors, adeno-associated viruses (AAVs) are the most applied in preclinical studies [21]. Due to their high loading capacity, biocompatibility, antigenicity, and lack of immune response, non-viral delivery systems based on cell-penetrating peptides (CPPs), micelles, polymers, and lipids have been commonly used in gene release studies [22,23,24,25]. Furthermore, ternary non-viral systems, for instance, constituted by polymers and/or peptides, have proven to be a valuable strategy to enhance payload encapsulation efficiency, thereby contributing to efficient gene delivery and expression [26,27,28].

CPPs have gained considerable interest in the gene therapy field due to their beneficial properties. CPPs are small (between 15 and 30 amino acids), and can be divided into arginine-rich and amphipathic peptides [29,30,31,32]. Because they have hydrophilic and hydrophobic domains, amphipathic peptides make it possible to formulate delivery systems that can encapsulate DNA and enable its membrane translocation and subsequent entry into cells [22,30]. Due to these characteristics, CPP-based vectors have demonstrated their ability to internalize in cells, and to deliver therapeutic molecules, in several studies [33,34,35,36].

Polymer-based systems are also widely explored as gene carriers, due to their favorable physicochemical properties, low toxicity, and tailorability [28,37,38,39]. In this context, one of the most used polymers has been polyethylenimine (PEI), due to its ability to transport different types of nucleic acids, regardless of their type and size [40,41]. Moreover, PEI has characteristics that allow it to go beyond the endosome/lysosome membrane [42]. PEI is a cationic polymer displaying a high positive charge density. This charge enables strong electrostatic interactions between its amine groups and DNA phosphate groups, resulting in the formation of nanoparticles in which the genetic material is mostly condensed within. PEI-based systems demonstrate high endosomolytic activity, critical to the potential success of mitochondrial gene delivery [43,44].

Currently, gene therapy with mitochondria as a therapeutic target is still a challenging strategy. However, some studies have demonstrated the feasibility of this type of therapy using mitochondrial targeting sequences (MTSs), and focused on the mitochondrial genes ATP6 (mitochondrially encoded synthase membrane subunit 6′) and ND4 (mitochondrially encoded NADH:ubiquinone oxidoreductase core subunit 4). Following this approach, their function was restored within their respective respiratory chain complexes and, consequently, the production of ATP was reestablished [45,46]. Other investigations revealed interesting therapeutic outcomes when addressing one of the most prevalent mitochondrial diseases—Leber’s hereditary optic neuropathy (LHON). The introduction of functional ND1 (mitochondrially encoded NADH dehydrogenase 1 protein) and ND4 genes, usually mutated in this type of disease, provided the replacement of the normal activity of complex I in the respiratory chain [47,48]. 

In this study, we aimed to find out the most suitable delivery system for mitochondrial gene therapy by comparing the efficacy of peptide- and polymer-based complexes. For this, CPP- and PEI-based compounds were designed/synthesized to complex the mitochondrial gene ND1’s plasmid DNA (pDNA). To ensure specific targeting of the mitochondria, moieties that confer affinity to this organelle were covalently coupled. In the case of PEI, dequalinium chloride (DQA) [49] was conjugated, resulting in the compound PEI–SA–DQA, whereas for CPPs, the MTS sequence [50] was added. DQA—a lipophilic cation—and especially its vesicular form (DQAsomes), has been demonstrated to selectively accumulate in the mitochondrial matrix [51,52,53]. To overcome the lack of stability exhibited by DQAsomes under conditions of low temperature and/or high salt concentration, researchers have conceived DQA-based carriers for payload release [53,54,55]. In this context, polymer–DQA delivery systems have been developed and optimized for mitochondrial targeting, leading to great advances in drug delivery to mitochondria [53,56,57,58].

Furthermore, as PEI–SA–DQA revealed a low ability to complex pND1—even at very high N/P ratios—TAT (the transcriptional activator protein in HIV-1—an 11-amino-acid peptide with 6 arginine and 2 lysine residues) was additionally included in PEI–DQA/pND1 complexes to complex pND1. These PEI-based ternary complexes were developed at various N/P ratios and adequately characterized. The biocompatibility profile was evaluated, and in vitro studies were carried out to assess the capacity of the developed complexes to reach the mitochondria. The physicochemical properties exhibited by the novel delivery systems, together with their ability to target the mitochondria and promote transgene expression, confer them with promising applicability as carriers for mitochondrial gene therapy. In addition, the comparison between various complexes based on CPPs and PEI revealed differences in their physicochemical properties, with repercussions on the capacity for mitochondrial targeting, gene delivery, and protein expression. This work demonstrated the efficacy of both peptide- and polymer-based delivery systems for mitochondrial targeting and mitochondrial gene expression. Furthermore, our study draws a comparison between peptides and polymers to reveal the most adequate delivery system to promote mitochondrial targeting and functional protein production, contributing to improvements/advances in the design of pDNA complexes for mitochondrial gene expression.

## 2. Materials and Methods

### 2.1. Materials

The following reagents were obtained from Sigma-Aldrich (St. Louis, MO, USA): trifluoroacetic acid (TFA), piperidine, oxyma, diisopropyl carbodiimide (DIC), Fmoc-amino acids, dimethylformamide (DMF), diisopropylethylamine (DIEA), dichloromethane (DCM), acetonitrile, and diethyl ether. AmphiSpheres 40™ resin was acquired from Agilent Technologies (Les Ulis, France). The Peptide Synthesizer Liberty Blue HT12™ was obtained from CEM (Matthews, NC, USA). The HPLC Pumps (321) and the FC 204 Fraction Collector were purchased from Gilson. The LKB-REC 102 was obtained from Pharmacia (Stockholm, Sweden). The HPLC System (Waters Alliance 2695) was obtained from Waters Corporation. The dialysis against water was carried out using 3k MCWO Dialysis Tubes (Spectrum laboratories Inc. Rancho Dominguez, CA, USA). The lyophilization was performed with a FreeZone 1 L Laboratory Lyophilizer (LABCONCO). DAPI was purchased from Invitrogen (Carlsbad, CA, USA), and MitoTracker Orange CMTMRos from Molecular Probes (Leiden, The Netherlands). TAT (47–57) peptide (YGRKKRRQRRR), chemically synthesized, was supplied as a lyophilized powder from Biomatik (Cambridge, ON, Canada). Commercial branched polyethylenimine (PEI) with average Mw of 10 kDa and 25 kDa, fluorescein isothiocyanate (FITC), succinic anhydride (SA), and N-hydroxysulfosuccinimide (NHS) were obtained from Sigma-Aldrich. Agarose and GreenSafe Premium were obtained from NZYTech Lda (Lisbon, Portugal). HeLa cancer cells were purchased from Invitrogen (Carlsbad, CA, USA), and human dermal fibroblasts (NHDF, Ref. C-12302, cryopreserved cells) were obtained from PromoCell (Heidelberg, Germany). 

All solutions for the preparation of the peptide-based systems were freshly prepared by using ultrapure-grade water, purified with a Milli-Q system from Millipore (Billerica, MA, USA). 

In this study, two different plasmids were used: the recoded plasmid DNA encoding green fluorescent protein (pGFP) (5.9 kbp)—a plasmid developed for exclusive mitochondrial translation, a kind gift from Dr. Diana Lyrawati [51]—and the plasmid pCAG-GFP-ND1 (pND1) (5.4 kbp), developed previously by our team through the cloning of the mitochondrial NADH dehydrogenase 1 protein-encoded gene (mtND1) in the pDNA vector. All details concerning gene cloning and plasmid production can be consulted elsewhere [59]. 

### 2.2. Methods

#### 2.2.1. Synthesis of Peptides and PEI–DQA

The peptides used in this work were synthesized according to the protocol described in a previous work [18]. The synthesis of the PEI–DQA polymer was carried out in two steps: The first step was the synthesis of PEI–SA. To the solution of PEI (10 and 25 kDa) in DMSO, succinic anhydride (molar ratio of PEI: SA = 1:10, 1:25, respectively) was added. The reaction mixture was stirred at room temperature for 24 h. The solvent was evaporated from the crude reaction mixture and dialyzed for 48 h in water using cellulose ester membranes. The dialysate was lyophilized to obtain a fluffy white powder at a yield of ~57%. In the second and final step of this synthesis, 200 mg of PEI–SA was dissolved in 5 mL of DMF, and EDC, NHS, and triethylamine solution were slowly added dropwise under vigorous stirring for 2 h. Then, 77.18 mg of dequalinium was dissolved in 2 mL of DMF, and this solution was added dropwise into the vigorously stirred solution described above. Thereafter, the reacting mixture was evaporated by rotovap, and 3 mL of distilled water was added to completely dissolve the product. The mixture was dialyzed (3 k MWCO membrane) against distilled water for 24 h. Finally, the product was lyophilized for 24 h. The average reaction yield was ca. 65%.

#### 2.2.2. Nuclear Magnetic Resonance Spectroscopy (NMR)

A proton nuclear magnetic resonance spectroscope (Bruker spectrometer-300 MHz, Bruker, Billerica, MA, USA) was used to examine the conjugate’s chemical structure in CDCl_3_ solution at 25 °C. The conjugates dissolved in CDCl_3_ (10 mg/mL) were measured with 64 scans.

#### 2.2.3. Formulation of Peptide/pDNA and PEI–DQA/TAT/pDNA Complexes

Peptide-based complexes were formulated according to the procedure described in a recent publication by our team [18]. 

PEI–DQA, TAT, and plasmid DNA stock solutions were prepared in sodium acetate buffer (0.1 mM sodium acetate/0.1 M acetic acid, pH 4.5) at a concentration of 0.5 mg/mL for PEI–DQA and TAT solutions, and 100 μg/mL for pDNA solutions. In ternary complexes, the determination of the N/P ratio was established by considering the proportion of charges, individually, for PEI or TAT in relation to pDNA. This parameter was defined as the molar ratio of the amine groups in the polymer or peptide—which represent the positive charges—to negatively charged phosphate groups in the pDNA. Different concentrations of PEI–DQA and TAT (50 μL) were added, drop by drop, to 150 μL of pDNA under vortex for 1 min. The mixture was left for equilibration for 30 min at room temperature to promote the formation of complexes. Thereafter, the formed complexes were centrifuged (12,000 rpm) for 20 min, and the pellet containing the complexes was recovered. The amount of non-complexed pDNA on the supernatants was visualized by horizontal electrophoresis for 30 min under 120 V in 1% agarose gel stained with GreenSafe Premium. Samples were analyzed under ultraviolet (UV) light using a FireReader Imaging System (UVITEC, Cambridge, UK). 

The ability of the systems to complex pND1 was also evaluated by quantifying the intensity of the bands of the respective agarose gels, and compared with the band intensity of the initial pND1 sample of each gel. The quantification of bands’ intensity was performed by densitometry using Image Lab software version 6.1. The percentage of complexation capacity (CC) was calculated according to the following formula:

(1) $$\mathrm{Complexation}\;\mathrm{capacity}\;(\%)=100-\left(\frac{\mathrm{Band}\;\mathrm{intensity}}{\mathrm{Band}\;\mathrm{intensity}\;\mathrm{of}\;\mathrm{initial}\;\mathrm{pND1}}\times 100\right)$$

#### 2.2.4. Determination of Size and Surface Charge

The average size and the surface charges exhibited by PEI–DQA/TAT/pDNA complexes were both inferred by dynamic light scattering (DLS) in a Zetasizer Nano ZS device (Malvern Instruments, Malvern, UK), at 25 °C. The pellet with the complexes was suspended in 5% glucose with 1 mM NaCl. For size determination, a He–Ne laser at 633 nm with non-invasive backscatter (NIBS) was applied, while zeta potential values were measured by electrophoretic light scattering optics with an M3-PALS laser (phase analysis light scattering). Data were considered from 3 independent measurements, each of which was performed with 12 runs. Malvern Zetasizer software v 6.34 was employed to analyze the set of results.

#### 2.2.5. Cell Culture

HeLa cells were grown in Dulbecco’s modified Eagle’s medium with Ham’s F12 Nutrient Mixture (DMEM-F12) and L-glutamine supplemented with 0.5 g/L sodium bicarbonate, 1.10 g/L HEPES, 10% heat-inactivated fetal bovine serum (FBS), and 1% (*v/v*) of a mixture of penicillin (100 µg/mL) and streptomycin (100 µg/mL). Cells were maintained in a 5% CO_2_ humidified atmosphere, at 37 °C until confluence was achieved.

#### 2.2.6. Cytotoxicity Evaluation

The cytotoxicity profile of PEI–DQA/TAT/pND1 complexes was monitored in HeLa cells by MTT (3-[4,5dimethyl-thiazol-2-yl]-2,5-diphenyltetrazolium bromide) assay. Cancer cells were seeded in a 96-well plate, at a density of 1 × 10^4^ cells/well, and were grown at 37 °C in a 95% O_2_/5% CO_2_ humidified atmosphere. Complexes (100 µL) were first resuspended in serum-free DMEM medium, and then applied to the well plates for 6 h. The medium was changed to end the transfection process. After incubation for periods of 24 h and 48 h, the redox activity was evaluated by MTT reduction. Measurements of absorbance at 570 nm were performed on a Bio-Rad Microplate Reader Benchmark. The medium without cells was settled as zero absorbance and considered for spectrophotometer calibration. Non-transfected cells were used as positive controls, while ethanol-treated cells were used as negative controls. The relative cell viability (%) compared to control wells was calculated by [A] test/[A] control × 100, where [A] test is the absorbance of the test sample and [A] control is the absorbance of the control sample. All measurements were performed in triplicate.

#### 2.2.7. Fluorescence Confocal Microscopy

##### FITC Plasmid Labeling

For the preparation of FITC-labelled pDNA, 2 µg of pDNA, 2 μL of FITC (in sterile anhydrous dimethyl sulfoxide, 50 mg/100 µL), and 81 μL of labeling buffer (0.1 M sodium tetraborate, pH 8.5) were mixed. The mixture was stirred for 4 h at room temperature and in the dark. Two-and-a-half volumes of 100% ethanol (212.5 μL) and one volume of 3 M NaCl (85 μL) were added. FITC pDNA samples were incubated at −20 °C overnight. Thereafter, they were centrifuged at 4 °C for 30 min, and the pellet was washed with ethanol (75%).

##### Live Cell Imaging

The cellular internalization and mitochondrial targeting capacity of PEI–DQA/TAT/FITC–pND1 complexes were monitored by confocal laser scanning microscopy (CLSM). HeLa cells (2 × 10^3^) were grown in µ-slide 8-plate wells until 50–60% confluence was attained. Cell nuclei and mitochondria were stained with DAPI and MitoTracker Orange dye, respectively. The complete medium was replaced with a serum-free culture medium 12 h before transfection; 1 µg of complexed FITC–pND1 was added to each well. Images of transfected cancer cells were acquired after 6 h. The LSM 710 confocal microscope (Carl Zeiss SMT, Inc., Oberkochen, Germany) was used to observe real live transfection, under a 63× oil immersion objective. Images were analyzed with LSM software (Carl Zeiss SMT, Inc., Oberkochen, Germany). Throughout the study, HeLa cells were kept at 37 °C with 5% CO_2_. All images were acquired using the same parameters, with the laser and filters corresponding to the respective DAPI (445/450 nm), FITC (525/550 nm), and MitoTracker (555/580 nm) dyes. After the acquisition, the images were processed under the same conditions and parameters using ImageJ software.

#### 2.2.8. Reverse Transcription Polymerase Chain Reaction (RT-PCR)

Reverse transcription polymerase chain reaction (RT-PCR) was used to qualitatively analyze mRNA GFP expression. HeLa cells were seeded in 12-well plates at a density of 2 × 10^5^ cells/well. The medium was removed 24 h after transfection, and cells were washed with PBS. Untreated cells were used as controls. The cells were lysed with TRIzol (250 μL/well), incubated for 5 min at room temperature, chloroform was added and the mixture stirred to promote RNA extraction. The samples were incubated for 10 min at room temperature and then centrifuged at 10,000 rpm, at 4 °C, for 15 min. To ensure RNA precipitation, the aqueous phase was withdrawn and 125 μL of ice-cold isopropanol was added. Another centrifugation cycle (10,000 rpm, 4 °C, 15 min) was performed; 125 μL of 75% ethanol in DEPC water was added to the obtained pellet to remove the organic compounds. After centrifugation, 20 μL of DEPC water was added to rehydrate the pellet, and samples were quantified using a NanoPhotometer™. In complement, electrophoresis analysis on agarose gel (1%) was carried out. The “Xpert cDNA Synthesis Kit” from GRiSP (GRiSP, Porto, Portugal) was utilized for the cDNA synthesis. All of the instructions provided by the manufacturer were followed. The amplification of cDNA was performed through the addition of 10 μL of RNase-free water, 1 μL of reverse primer (5′-CGTTCTTGTACGTAGCCTTC-3′), 1 μL of forward primer (5′-CTGCACCACCGGAAAACTCC-3′), 12 μL of Speedy Supreme NZYTaq 2× Green Master Mix (NZYTech, Lisbon, Portugal), and 1 μL of cDNA, in each PCR reaction. After homogenization of the samples, they were placed in a T100™ thermal cycler (Bio-Rad Laboratories, Inc., Hercules, CA, USA). The following reaction conditions were established: denaturation (94 °C for 2 s), annealing (57 °C for 5 s), and extension (72 °C for 5 s) for 35 cycles. Agarose gel electrophoresis was used to analyze the PCR products. The visualization was performed on a UV FireReader Imaging System (UVITEC, Cambridge, UK).

#### 2.2.9. Mitochondrial Isolation

Mitochondria were isolated from other cellular organelles with the Mitochondria Isolation Kit for Cultured Cells (Thermo Fisher Scientific Inc., Rockford, IL, USA). This kit ensures the separation of mitochondria with high purity and yield. The instructions provided by the manufacturer were followed. Briefly, HeLa cells (1 × 10^4^) were transferred to Falcon tubes, and 800 µL of Reagent A was added to the cells, followed by incubation on ice for 2 min. Then, 10 µL of Reagent B was added, and the cells were vortexed for 10 s at maximum speed, incubated on ice for 5 min, and vortexed again at maximum speed every minute for 10 min. After this, Reagent C (800 µL) was added, and samples were centrifuged (20,000× *g*) for 10 min at 4 °C. Then, 500 µL of Reagent C was added to the resultant pellet containing the mitochondria. Final centrifugation at 12,000× *g* for 5 min was performed, and the supernatant was discarded. The obtained pellets full of mitochondria were resuspended in 50 µL of ice-cold PBS and mixed with 500 µL of carbonate buffer (fresh cold 0.1 M Na_2_CO_3_).

#### 2.2.10. Protein Quantification

The ND1 protein produced by transfection of HeLa cells with the developed systems was identified using an ND1 ELISA kit (Biomatik, EKL54820, Wilmington, DE, USA), following the procedure described by the manufacturer. ND1 was quantified by a sandwich enzyme immunoassay. Transfected HeLa cells with different peptide- or polymer-based complexes were lysed following standard cell lysis methods. Cells were detached with trypsin and centrifuged. They were then washed three times in cold PBS, resuspended in PBS, and ultrasonicated 4 times. After this procedure, HeLa cells were centrifuged at 15,000× *g* for 10 min at 4 °C. The manufacturer provided reagent solutions that were used according to instructions. In summary, Reagent A was added to each well and incubated for 1 h at 37 °C, followed by incubation with Reagent B. Thereafter, TMB substrate solution was added to each well, and samples were incubated for 20 min at 37 °C in the dark. A sample displaying a yellow color was obtained after the addition of Stop Solution. The content of ND1 protein was inferred by measuring the absorbance in a microplate reader at 450 nm. 

#### 2.2.11. Statistical Analysis

The normality of the distribution of sample data was evaluated by running appropriate tests, such as the D’Agostino–Pearson omnibus. The statistical analysis performed was a one-way or two-way analysis of variance (ANOVA), followed by Bonferroni’s multiple comparison test. A *p*-value below 0.05 was considered statistically significant. Data analysis was conducted with GraphPad Prism v.8.01 (GraphPad Software, Inc., San Diego, CA, USA).

## 3. Results and Discussion

### 3.1. Synthesis and Characterization of PEI–SA, and PEI–DQA

**Figure 1:**
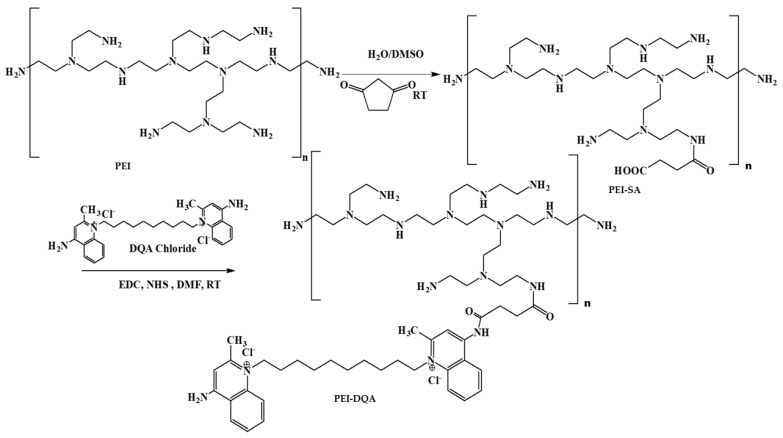
Scheme illustrating the synthesis of the polymer PEI–DQA. RT: room temperature.

**Figure 2:**
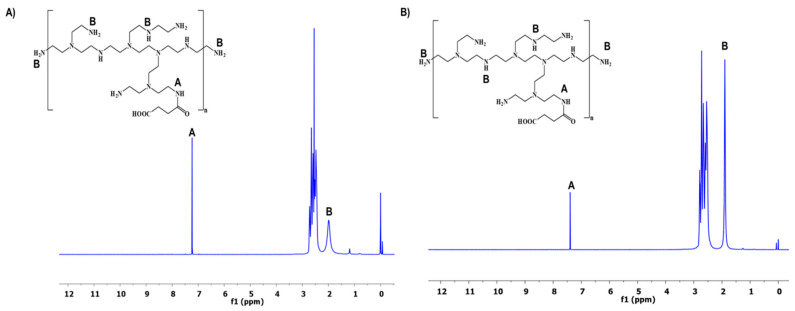
^1^H NMR spectra of the intermediate PEI–SA: PEI-10 kDa (**A**) and PEI-25 kDa (**B**).

**Figure 3:**
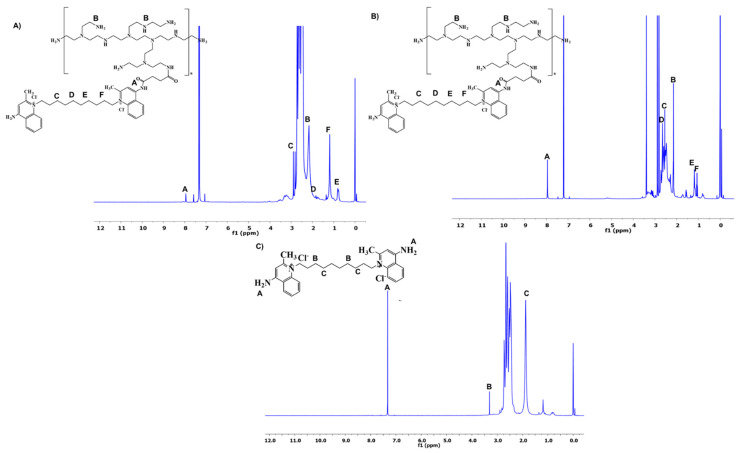
^1^H NMR spectra of PEI–DQA: PEI (10 kDa) (**A**) and PEI (25 kDa) (**B**); the spectrum of DQA (**C**).

The synthesis of PEI conjugates was carried out following the general scheme depicted in Figure 1. The amine-terminated PEI was succinated to further react with DQA. The terminal carboxylic acid group of succinic acid provided the attachment point with DQA via the acid–amine coupling reaction. The reaction conditions produced optimal yield at each step. The analysis of synthesized PEI derivatives was carried out using spectroscopic methods. The ^1^H NMR spectra of PEI-SA (10 and 25 kDa) are shown in Figure 2A,B, respectively. In both spectra, broad multiplet peaks were observed at δ3.12–3.42 ppm, corresponding to the methylene groups present in the primary amine group of PEI, along with the introduced succinic substituent ((PEI)–(CH_2_)_2_–NH–CO–(CH_2_)_2_–COOH). For DQA conjugates, the characteristic signals of the aromatic ring protons were present at δ7.25–7.35 ppm (Figure 3A,B). The peak located at δ3.32 ppm was identified as benzyl–CH_2_ protons. The methylene moiety had a broad multiplet at δ3.63–3.72 ppm. The successful conjugation in PEI–DQA (10 and 25 kDa) was confirmed from ^1^H NMR. Figure 3C shows the DQA spectrum. Fourier-transform infrared (FTIR) spectroscopy was carried out to confirm the success of the modification. Figure S1, available in the Supplementary Materials (SM), shows the FTIR spectra of PEI–DQA (10 kDa and 25 kDa) (A) and the FTIR spectra of DQA (B). The signals at 3300 cm^−1^ and 2822–2530 cm^−1^ indicated the N–H stretching and aliphatic C–H stretching vibrations, respectively, in all PEI–SA conjugates. The PEI–DQA (10 and 25 kDa) displayed a broad peak at 1720 cm^−1^ as the stretching vibrations of the C=O moiety. Similarly, the C-O-C (1228 cm^−1^) and N-H (3210 cm^−1^) stretching vibrations were observed in the PEI–DQA conjugates. The chromatogram obtained via gel permeation chromatography (GPC) is represented in Figure S2, available in the SM. The peak shift on the left due to the change in retention time of the polymer and polymer conjugates indicated the increase in molecular weight. According to the molecular weights of the conjugates obtained through GPC analysis (Table S1 in the SM), ~11 molecules of SA were attached to PEI (10 kDa), which conjugated ~9 molecules of DQA. Similarly, PEI (25 kDa) was attached to ~15 molecules of SA, which resulted in ~13 molecules of DQA attachment. The critical micelle concentration (CMC) was observed to be 12.5 µg/mL for PEI–DQA (10 and 25 kDa) conjugates. The reduced CMC showed that PEI–DQA conjugates can readily develop a core–shell structure in an aqueous environment (Figure S3 in SM).

### 3.2. pDNA Complexation Capacity

The formulation of PEI–DQA/TAT/pND1 systems was elaborated using a dropwise precipitation method. First, PEI–DQA solution was added to pDNA solution for 1 min, after which TAT peptide solution was added. Electrostatic interactions are the main force that allows the encapsulation of pDNA by PEI–DQA and TAT. These interactions occur due to the negative charges on pND1 that bind to the positive charges on both PEI and TAT, forming nanoscale systems. The interaction strength is dependent on the N/P ratio considered at the formulation step, and the higher the ratio, the greater the availability of positive charges to interact with pND1. Moreover, in previous research by our team, the cytotoxicity of TAT/pDNA complexes was evaluated as a function of the N/P ratio on both fibroblasts and HeLa cells [26]. The results demonstrated that TAT/pDNA complexes formulated at N/P ratios of 1, 2, 4, 8, and 10 are all biocompatible. After incubation with TAT-based complexes, a moderate increment in the cellular viability was observed as the N/P ratio increased. 

**Figure 4:**
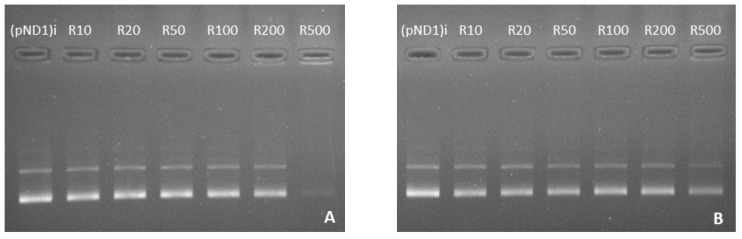
Agarose gel electrophoresis of the initial pND1 solution ((pND1)i) and supernatants resulting from the formulation of PEI–DQA (10 kDa)/pND1 systems (PEI–DQA N/P = 10, 20, 50, 100, 200, and 500) (**A**) and PEI–DQA (25 kDa)/pND1 systems (PEI–DQA N/P = 10, 20, 50, 100, 200, and 500) (**B**).

**Figure 5:**
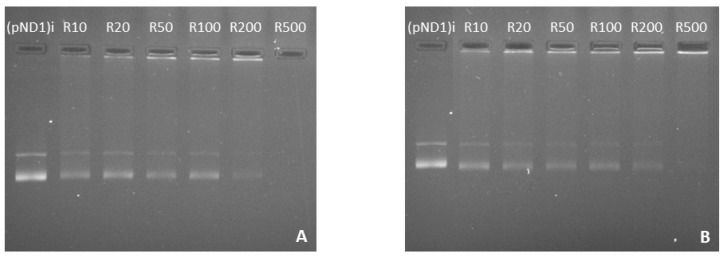
Agarose gel electrophoresis of the initial pND1 solution ((pND1)i) and supernatants resulting from the formulation of PEI–DQA (10 kDa)/TAT/pND1 systems (PEI–DQA N/P = 10, 20, 50, 100, 200, and 500, and TAT N/P = 1) (**A**) and PEI–DQA (25 kDa)/TAT/pND1 systems (PEI–DQA N/P = 10, 20, 50, 100, 200, and 500, and TAT N/P = 1) (**B**).

**Figure 6:**
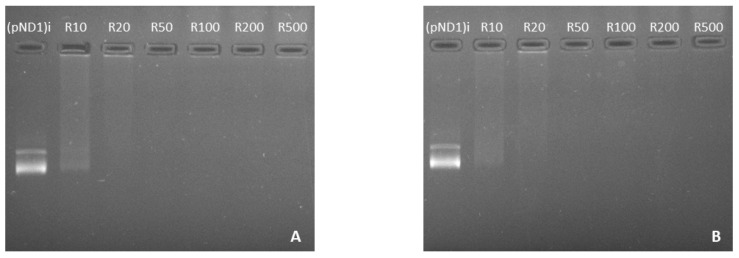
Agarose gel electrophoresis of the initial pND1 solution ((pND1)i) and supernatants resulting from the formulation of PEI–DQA (10 kDa)/TAT/pND1 systems (PEI–DQA N/P = 10, 20, 50, 100, 200, and 500, and TAT N/P = 2) (**A**) and PEI–DQA (25 kDa)/TAT/pND1 systems (PEI–DQA N/P = 10, 20, 50, 100, 200, and 500, and TAT N/P = 2) (**B**).

**Figure 7:**
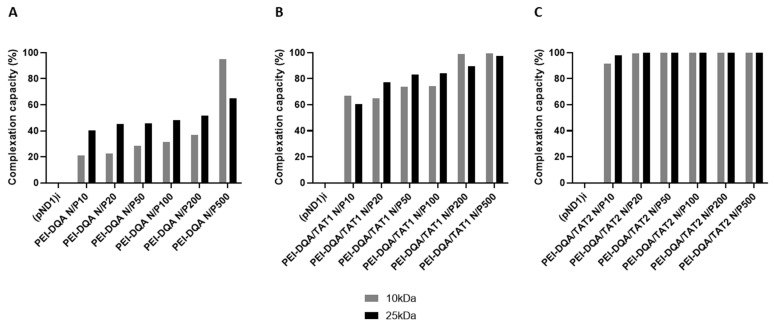
pND1 complexation capacity (CC) of PEI–DQA (10 and 25 kDa)/pND1 (**A**), PEI–DQA (10 and 25 kDa)/TAT(N/P1)/pND1 (**B**), and PEI–DQA (10 and 25 kDa)/TAT(N/P2)/pND1 (**C**) complexes calculated from the band intensity of the agarose gels of Figure 4, Figure 5 and Figure 6 respectively.

Here, the complexation capacity of the systems and the influence of the N/P ratio were evaluated using agarose gel electrophoresis. The ratios of PEI–DQA to pND1 used were 10, 20, 50, 100, 200, and 500, and the ratios of TAT to pND1 were 1 and 2. Results are shown in Figure 4, Figure 5 and Figure 6. In Figure 4 we can see that the 10 kDa and 25 kDa PEI–DQA systems had a low capacity to encapsulate pND1, even at the highest ratios (100:1 and 200:1). Figure 5 concerns 10 kDa and 25 kDa PEI–DQA systems to which TAT was added, where N/P = 1. By analyzing the images, we found that all formulated systems exhibited encapsulation capacity to some extent, compared to the initial pND1 sample. However, only systems prepared at a 500:1:1 ratio of both PEIs demonstrated high encapsulation capacity. As PEI is a compound known to have considerable cytotoxicity at high concentrations, the need arose to try to improve encapsulation by keeping PEI ratios as low as possible. Following this, we doubled the TAT ratio, which resulted in a great improvement in pND1 encapsulation for all formulated systems, maintaining PEI ratios (Figure 6). The prepared systems presented a high efficiency of pND1 encapsulation, being able to neutralize its negative charges; thus, there were no bands in the agarose gel. Moreover, pND1 CC (%) was calculated from the band intensities of the agarose gels, as described in the Materials and Methods section; the results are shown in Figure 7. PEI (10 or 25 kDa)–DQA/pND1 complexes displayed poor ability to complex pND1 at lower ratios, and it was found that CC varies with the N/P ratio (Figure 7A). It seemed that an increase in the N/P ratio led to high CC values. Additionally, in general, PEI (25 kDa)–DQA/pND1 complexes condensed pND1 to a higher extent when compared to the capacity exhibited by the corresponding PEI (10 kDa) complexes. The incorporation of TAT peptide into PEI (10 or 25 kDa)–DQA/pND1 complexes significantly increased their capacity to condense pND1 (Figure 7B,C). Considerably high CC values were obtained for all N/P ratios investigated. At an N/P ratio of TAT/pND1 of 2, practically all complexes possessed a CC of around 100%. The exception was PEI (10 kDa)–DQA/TAT/pND1 prepared at the lowest ratio (Figure 7C). From this, there was no need to consider TAT/pND1 ratios higher than 2. In this work, we also intended to compare these PEI-based systems with previously studied systems based on peptides that also have sequences to confer specificity for mitochondrial targeting [18]. Comparing the pND1 encapsulation ability of PEI–DQA/TAT/pND1 complexes with that displayed by peptide-based complexes, PEI-based complexes showed lower efficiency for pND1 encapsulation, since to encapsulate the same amount of pND1 higher N/P ratios were required (N/P ratios of 10 versus N/P ratios of 0.5 and 1). This lower capacity can be explained by the decrease in the amines available in PEI due to the addition of DQA to the polymer, reducing the charges available to interact with pND1. Nevertheless, our study proceeded in analyzing the properties of PEI–DQA/TAT/pND1 systems and their potential for mitochondrial targeting/gene delivery.

### 3.3. Characterization of PEI–DQA/TAT/pND1 Complexes

**Figure 8:**
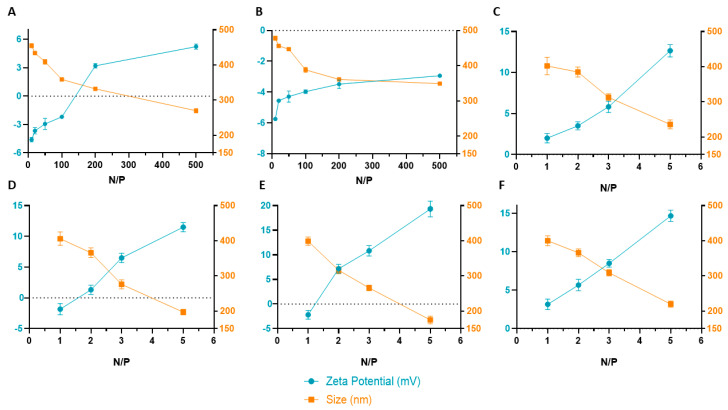
Average zeta potential and mean size displayed by PEI–DQA (10 kDa and 25 kDa)/TAT/pND1 (**A**,**B**), CpMTP/pND1 (**C**), MTS-WRAP1/pND1 (**D**), MTS-WRAP5/pND1 (**E**), and MTS-(KH)_9_/pND1 (**F**) complexes.

After verifying the complexation capacity of the produced systems, these complexes were characterized by DLS in terms of size and surface charge. The results are shown in Figure 8, corresponding to data from PEI–DQA (10 kDa)/TAT/pND1 and PEI–DQA (25 kDa)/TAT/pND1 complexes. In addition, Table S2 (in SM) presents all of the obtained values of size and zeta potential for both polymer- and peptide-based systems. The results of surface charge measured in zeta potential (mV) for systems with PEI–DQA of 10 kDa revealed a slightly negative overall charge. A decrease in this negative charge with the increase in the N/P ratio was also observed. The complexes prepared with PEI–DQA (25 kDa), for the lowest N/P ratios under study, showed a charge very close to 0 mV, while for N/P ratios of 200:2:1 and 500:2:1 they presented positive zeta potential values (+3.2 and +5.2 mV, respectively). This low global charge can be explained by the presence of DQA, which presents a neutral charge [49], decreasing the influence of positive charges of PEI on the surface of complexes. The main contribution to zeta potential comes from the inclusion of TAT. The surface charge of TAT/pND1 complexes conceived at an N/P ratio of 2 was found to be around +2.3 mV. When compared to these later data, results regarding the physicochemical properties exhibited by the peptide/pND1 complexes [18] presented in the previous publication demonstrated that peptide-based complexes displayed a more positive surface charge, with zeta potential values of up to +20 mV for an N/P = 5, while the highest value for PEI–DQA/TAT/pND1 complexes was +5.2 mV for N/P = 500. 

Evaluation of the size of the formulated polyplexes revealed a difference between complexes formulated with PEI of 10 kDa and 25 kDa. For the same N/P ratio, average sizes were smaller in systems prepared with 25 kDa of PEI. The effect of the N/P ratio was also evident within the same type of system—as the ratio increased, there was a decrease in the size of complexes. This evidence was due to the greater capacity of high-molecular-weight PEI to encapsulate pND1, due to high charge density that led to stronger electrostatic interactions. The sizes of all complexes were less than 500 nm, which may have facilitated cell internalization. The sizes evidenced by these systems were superior to the sizes displayed by peptide-based complexes formulated in our previous study [18]. Comparing the results, we previously found that peptide-based complexes had sizes around 150–200 nm for the highest ratios, while in this study PEI–DQA/TAT/pND1 complexes exhibited sizes around 350 nm. Thus, we can infer that peptide/pND1 complexes presented a greater capacity to condense pND1 and formulate smaller complexes. Another relevant characterization parameter is the PdI, which allows us to assess the distribution of sample sizes. PdI values close to 0.01 reveal monodisperse samples; between 0.5 and 0.7 are considered polydisperse particles and a value above 0.7 is indicative of broad particle size distribution [60]. PdI data are presented in Table S2, available in the SM. Analyzing this table, we verified a PdI between 0.4 and 0.6, with an effect on the N/P ratio. Lower ratios corresponded to polydisperse samples, while higher ratio complexes were monodispersed. Compared to PdI values obtained for peptide-based complexes, the difference was minimal. PdI for peptide/pND1 systems was around 0.3, with polydisperse samples found for smaller ratios.

Complexes of MTS-CPP/pND1 and CpMTP/pND1 were previously published [18]. The values were calculated with data obtained from three independent measurements (mean ± SD, *n* = 3).

### 3.4. Cytotoxic Profile

**Figure 9:**
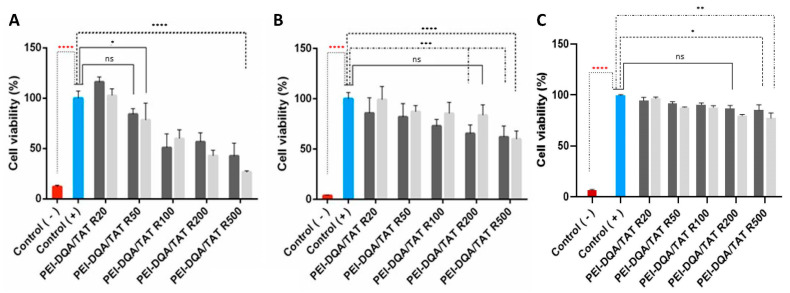
Cellular viability of HeLa cells after 24 h (**A**), 48 h (**B**), and 72 h (**C**) of incubation with PEI–DQA (10 kDa or 25 kDa)/TAT/pND1 complexes conceived at N/P ratios of 20:2:1, 50:2:1, 100:2:1, 200:2:1, and 500:2:1. Non-transfected cells were used as positive controls (100 % viability) and ethanol-treated cells were considered as negative controls (0 % viability). Statistical analysis was carried out using “one-way ANOVA” with data obtained from four independent measurements (mean ± SD, *n* = 4).

The cytotoxicity of formulated polymeric complexes was evaluated using the MTT assay in HeLa cells. To verify the safety of the complexes, the viability of HeLa cells was evaluated at 24 h, 48 h, and 72 h after transfection with the various systems under study. PEI–DQA (10 kDa or 25 kDa)/TAT/pND1 was used at N/P ratios of 20, 50, 100, 200, and 500, and TAT/pND1 N/P of 2:1. The results shown in Figure 9A–C correspond to 24, 48, and 72 h transfection, respectively. Non-transfected cells were used as positive controls (100% viability), while cells treated with 70% ethanol were used as negative controls (0% viability). Statistical analysis was performed with the positive controls for comparison. Systems formulated with high ratios demonstrated cytotoxicity when compared to the positive controls (N/P ratios of 100:2:1; 200:2:1, and 500:2:1). Polymeric complexes formulated at low ratios demonstrated biocompatibility (statistically not significant (n.s) versus positive controls). Therefore, these results showed the influence of the N/P ratio on the cytotoxicity of the developed systems. The increment in the N/P ratio led to an increased content of PEI amines, and this high cationic charge corresponded to high cytotoxicity. In this way, it can be pointed out that the N/P ratio can be viewed as a tailoring tool to adjust cellular cytotoxicity levels. At 24 h, the obtained data also demonstrated some differences between complexes formulated with PEI–DQA (10 kDa) and PEI–DQA (25 kDa). At 24 h and for the highest ratios, PEI–DQA (25 kDa) complexes were more cytotoxic than the ones based on PEI–DQA (10 kDa); there was a statistically significant difference (* *p* ≤ 0.05) for the comparison between these complexes at N/P ratios of both 200:2:1 and 500:2:1. This tendency, however, was not observed at 48 h. The cytotoxicity evidenced in complexes prepared at high ratios seemed to be more pronounced in the first 24 h when compared to that obtained at 48 h and 72 h after transfection. We can hypothesize that cells suffered an initial shock during the first 24 h of incubation, with a significant loss of their viability, after which they were able to recover over time, showing higher viability. However, a clear explanation for this phenomenon cannot be anticipated at this stage. For instance, at 72 h and at high N/P ratios, the difference from the positive controls was lower (* *p* ≤ 0.05 or ** *p* < 0.01 for PEI–DQA (25 kDa)/TAT/pND1 at 500:2:1 versus positive controls) than the difference found at 24 h (for high N/P ratio complexes, **** *p* < 0.0001 versus positive control) or 48 h (for most N/P ratio complexes, *** *p* < 0.001 versus positive control). 

For further in vitro studies, and due to their biocompatibility, lower N/P ratio PEI–DQA (10 kDa or 25 kDa)/TAT/pND1 complexes were selected. 

The biocompatibility of peptide-based complexes was analyzed and discussed elsewhere [18]. All peptides/pND1 were demonstrated to be non-toxic to HeLa cells at 24 h, with the obtained results being statistically non-significant (n.s) in relation to the positive controls. At 48 h, however, a decrease in biocompatibility was observed for all peptides/pND1 nanoparticles. Furthermore, the presence of the MTS sequence in the peptides seemed to influence the cytotoxic profile displayed by the complexes. MTS-peptide/pND1 carriers exhibited less cellular viability in relation to controls [18]. Comparing cytotoxicity results between polymer and peptide/pND1 complexes, it was verified that the latter complexes presented higher safety in terms of biocompatibility, making them far superior in relation to PEI–DQA/TAT/pND1 complexes. These results can be explained by the greater efficiency of peptides in encapsulating pND1, decreasing the need to use high N/P ratios.

### 3.5. Mitochondrial Targeting Ability

Over the past few years, researchers have pursued the goal of mitochondrial targeting and mitochondrial transgene expression for the treatment of mitochondrial disorders [52]. This effort has led to the development of efficient delivery systems with the ability of cellular internalization, followed by mitochondrial targeting [52]. Recently, our team has also contributed to this field with the conception of mitochondria-targeted peptide/pND1 complexes using a mitochondrial targeting sequence (MTS) coupled to peptides able to internalize nucleic acids such as MTS-WRAP1, MTS-WRAP5, and MTS-(KH)_9_ [18]. In this previous work, real live transfection of HeLa cells mediated by these peptide delivery vectors, monitored by fluorescence confocal microscopy, revealed that the complexes were easily taken up by cancer cells and accumulated at the site of the mitochondria [18], unequivocally demonstrating their mitochondrial targeting specificity. Among those peptide complexes, the ones based on the CpMTP peptide seemed to display higher cell penetration ability and targeting performance [18].

**Figure 10:**
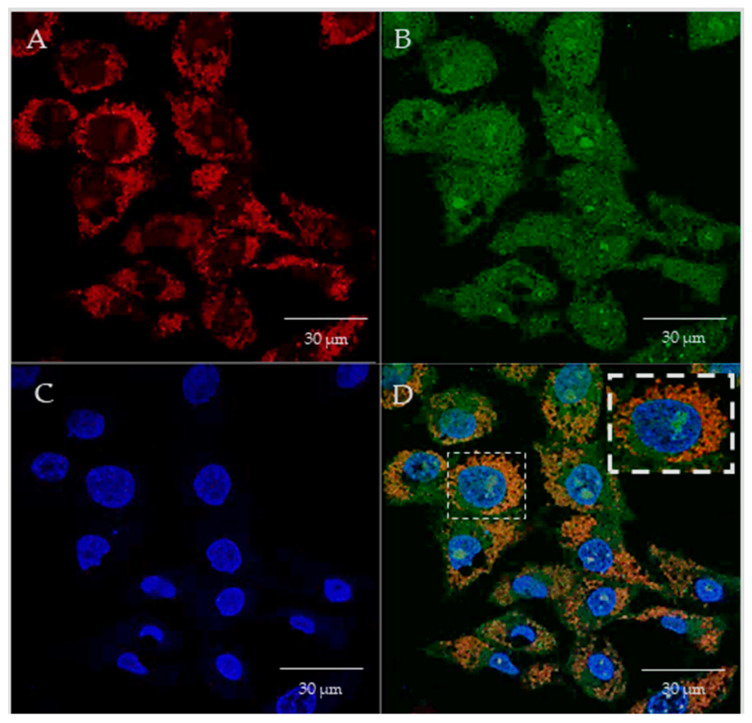
Cellular uptake and intracellular colocalization of PEI–DQA (10 kDa)/TAT/pND1 complexes formulated at an N/P ratio of 50:2:1. Mitochondria stained red by MitoTracker (**A**), green labeled pND1 (**B**), nuclei marked blue by DAPI (**C**), and merged image (**D**).

**Figure 11:**
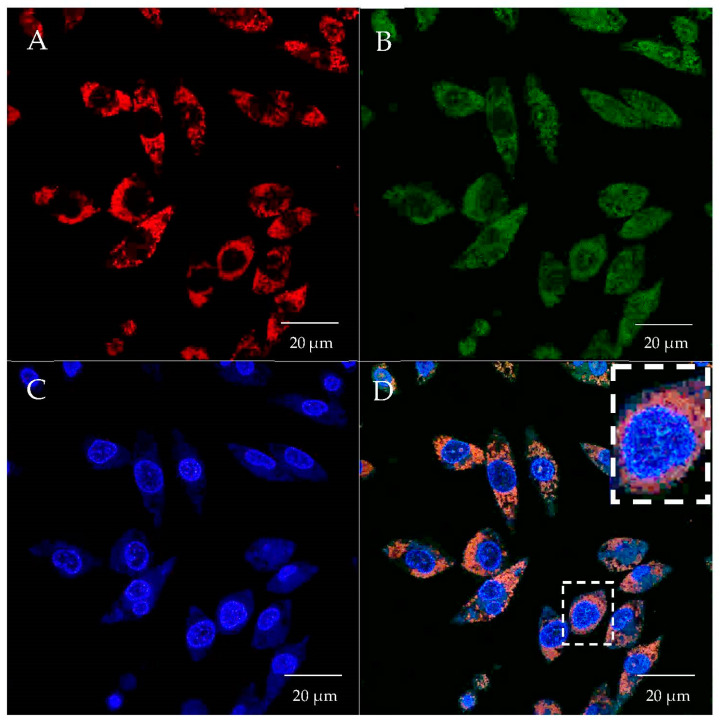
Cellular uptake and intracellular colocalization of PEI–DQA (25 kDa)/TAT/pND1 complexes formulated at an N/P ratio of 50:2:1. Mitochondria stained red by MitoTracker (**A**), green labeled pND1 (**B**), nuclei marked blue by DAPI (**C**), and merged image (**D**).

In the present study, we investigated the mitochondrial targeting capacity of various synthesized PEI–DQA/TAT/pND1 complexes in HeLa cells. In this sense, the cellular internalization and mitochondrial targeting ability of these polymeric complexes were assessed by confocal microscopy, using appropriate dyes to stain the nuclei and mitochondria, while pND1 was labeled with FITC. In this way, real live transfection of HeLa cells mediated by PEI–DQA (10 kDa or 25 kDa)/TAT/pND1 vectors, conceived at different N/P ratios, was monitored. The obtained cell images, at 6 h of transfection, are visualized in Figure 10 and Figure 11, corresponding to the cellular transfection mediated by PEI–DQA (10 kDa)/TAT/pND1 at an N/P ratio of 50:2:1 and PEI–DQA (25 kDa)/TAT/pND1 at an N/P ratio of 50:2:1, respectively. The images were obtained from a set of consecutive Z planes (Z-stacks; step size of 0.1 µm). In both presented figures, image A represents mitochondria labeled with MitoTracker, image B shows PEI–DQA (10 kDa or 25 kDa)/TAT/pND1 complexes, image C shows nuclei marked with DAPI, and image D corresponds to the merged image. From the green fluorescence visualized in B, we inferred that efficient transfection took place and polymeric complexes were internalized in HeLa cells. 

Moreover, after the uptake, the developed delivery complexes were targeted to the site of the mitochondria. Image D in both figures shows the specific accumulation of PEI–DQA (10 kDa or 25 kDa)/TAT/pND1 complexes in the mitochondria, demonstrating their mitochondria-specific targeting. We also evaluated the effect of the N/P ratio on the targeting capacity by performing the described microscopy study on HeLa cells using PEI–DQA (10 kDa or 25 kDa)/TAT/pND1 formed at a lower N/P ratio of 20:2:1. The collected images can be consulted in the SM (Figures S4 and S5). Very similar observations were made, revealing that both studied N/P ratios were adequate to develop polymeric complexes able to target the mitochondria. This targeting skill, displayed by the novel PEI-based complexes, represents a great asset to further studies on mitochondrial gene delivery and expression. Furthermore, as evidenced in the merged panel of Figure 10 and Figure 11, although the complexes were preferentially located in the mitochondria, some of the complexes were found in the nuclei (especially in the nucleoli).

### 3.6. Evaluation of Gene Expression

**Figure 12:**
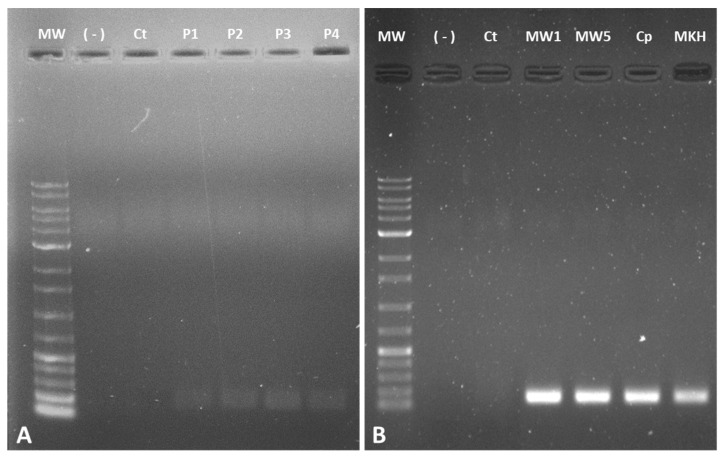
Analysis of GFP gene expression by RT-PCR after transfection of HeLa cells with (**A**) P1—PEI–DQA (10kDa)/TAT/pGFP, N/P of 20:2:1; P2—PEI–DQA (10 kDa)/TAT/pGFP, N/P of 50:2:1; P3—PEI–DQA (25kDa)/TAT/pGFP, N/P of 20:2:1; P4—PEI–DQA (25 kDa)/TAT/pGFP, N/P of 50:2:1; (**B**) MW1—MTS-WRAP1/pGFP, N/P of 5:1; MW5—MTS-WRAP5/pGFP, N/P of 5:1; Cp—CpMTP/pGFP, N/P of 5:1 and MKH—MTS-(KH)_9_/pGFP, N/P of 5:1. MW—molecular weight; (-)—PCR control; CT—non-transfected cells.

Gene expression was evaluated using recoded mitochondrial pGFP—a pDNA designed for specific expression in the mitochondria [51]. Polymeric and peptide/pGFP delivery systems were prepared at various N/P ratios, following the method described in the Materials and Methods section for pND1. Thereafter, HeLa cells were transfected with the formed complexes, and recoded GFP gene expression was evaluated by RT-PCR. Results obtained for PEI- and peptide-based complexes are shown in Figure 12A,B, respectively. Non-transfected cells were used as controls. The figures show agarose gel electrophoresis of amplification products of the mitochondrial GFP gene, with bands at expected sites. The results obtained for PEI–DQA (10 kDa or 25 kDa)/TAT/pGFP complexes, at N/P ratios of 20:2:1 or 50:2:1 (Figure 12A), demonstrated the efficacy of HeLa cells’ transfection and the presence of pGFP transcripts to a higher extent when compared to controls. Although the bands on the agarose gel were faint, it seemed that all considered polymeric complexes led to GFP expression in the mitochondria.

The RT-PCR results for peptide/pGFP systems (Figure 12B) demonstrated a clear detection of GFP mRNA in transfected cells, when compared to mRNA levels detected in control cells. In general, wide and intense bands were obtained for all peptide-based complexes. Since the total amount of RNA transcribed to cDNA was fully comparable in both polymeric and peptide systems, (see the Materials and Methods section for details), it seemed that the extent of gene expression was higher for the latter systems. Apparently, peptide-based systems seemed to be more promising for GFP expression in the mitochondria. We are, however, aware that RT-PCR results are merely qualitative, giving first evidence of gene expression and potentiating further analysis. To deeply infer the content of both gene and protein, more accurate assays are required.

### 3.7. Quantification of Protein

**Figure 13:**
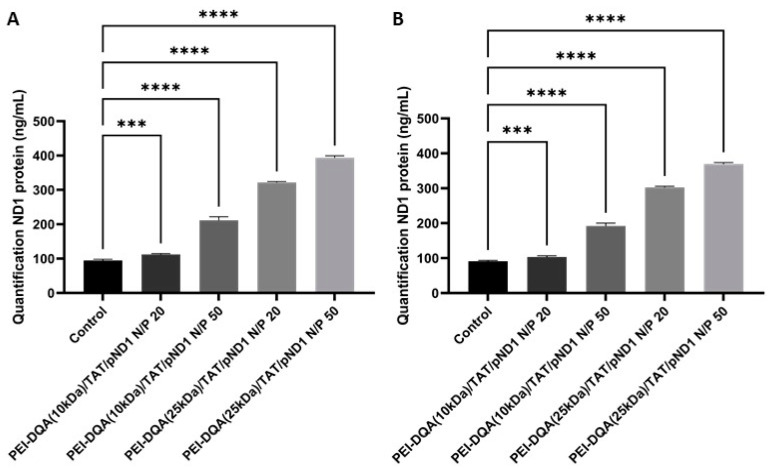

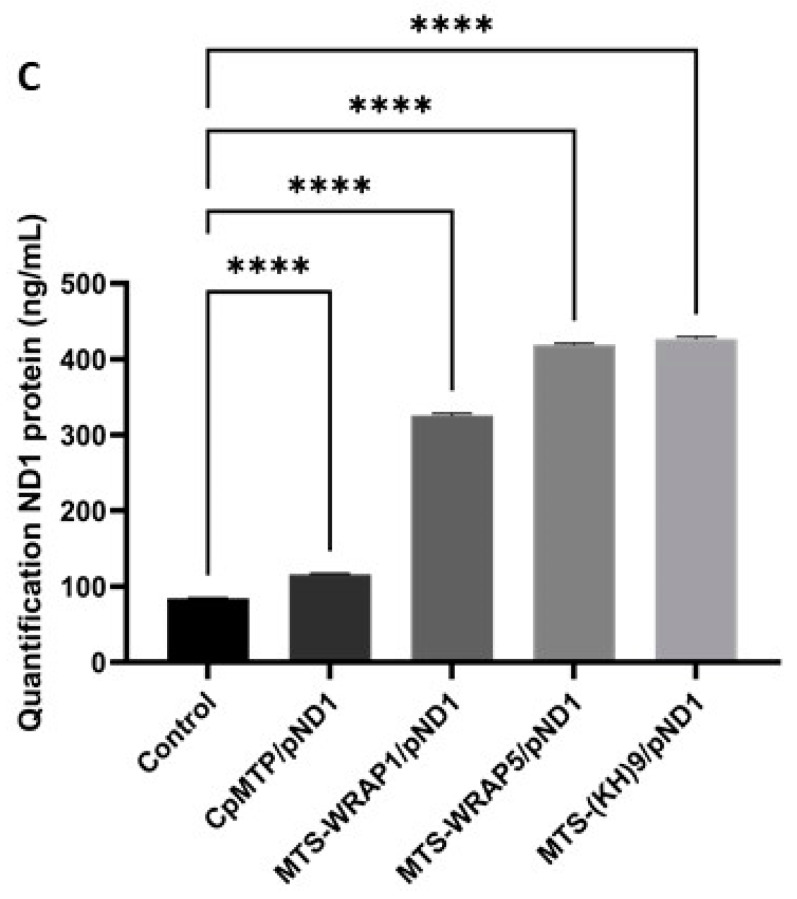
Quantification of ND1 protein 48 h after transfection of fibroblasts (**A**) and HeLa cells (**B**) with PEI–DQA (10 kDa)/TAT/pND1, N/P = 20:2:1; PEI–DQA (10 kDa)/TAT/pND1, N/P = 50:2:1; PEI–DQA (25 kDa)/TAT/pND1, N/P = 20:2:1; and PEI–DQA (25 kDa)/TAT/pND1, N/P = 50:2:1; and 48 h after transfection of HeLa cells (**C**) with CpMTP/pND1, MTS-WRAP1/pND1, MTS-WRAP5/pND1, and MTS-(KH)_9_/pND1—all complexes prepared at an N/P ratio of 5:1. Non-transfected cells were used as controls. Data were analyzed by one-way ANOVA. (*** *p* < 0.001 or **** *p* < 0.0001).

The evidence of gene delivery and expression in mitochondria instigated us to quantify the produced ND1 protein, after transfection mediated by polymeric or peptide/pND1 complexes. In this way, ND1 protein expression in the mitochondria of HeLa and fibroblast cells was measured by using an ELISA kit. Non-transfected cells served as controls. The results are shown in Figure 13. Control cells presented significant levels of ND1 protein, as ND1 is an endogenous mitochondrial gene. As observed, this amount can be increased by the transfection with the developed mitochondria-targeted/delivery systems. As shown in Figure 13A,B, both fibroblast (A) and HeLa (B) cells transfected with PEI–DQA/TAT/pND1 complexes demonstrated higher levels of ND1 protein when compared to controls (*** *p* < 0.001 or **** *p* < 0.0001). Moreover, it was observed that this increase in the production of ND1 protein was influenced by both the molecular weight of PEI and the N/P ratio. The results demonstrated that PEI (25 kDa) led to a higher amount of ND1 in comparison with the protein levels when transfection was mediated by complexes based on PEI (10 kDa), even at lower N/P ratios. Moreover, high-N/P-ratio systems demonstrated higher ND1 levels compared to lower ratio systems when using PEI of the same molecular weight. The results obtained for fibroblasts and HeLa cells were very similar (Figure 13A,B, respectively), evidencing a pattern independent of the cell type. 

Figure 13C showed ND1 content produced from the transfection of HeLa cells with peptide/pND1 complexes. After 48 h of transfection, and similar to polymeric systems, peptide complexes led to high ND1 levels when compared to control cells (**** *p* < 0.0001). Among peptide complexes, the CpMTP peptide was found to induce the production of the lowest amount of ND1. Cells transfected with MTS-WRAP1, MTS-WRAP5, and MTS-(KH)_9_ systems produced protein levels 3 to 4 times higher than non-transfected cells. 

Comparing PEI complexes with peptide-based systems, we found that peptides led to ND1 concentrations above 400 ng/mL (for MTS-WRAP5/pDN1 and MTS-(KH)9/pND1 complexes), while complexes based on PEI did not reach this protein concentration, even when using 10 times higher N/P ratios. Therefore, peptide/pND1 complexes seemed to be more efficient in both gene and protein expression in mitochondria than the polymeric complexes. This assumption also correlated well with previously obtained data regarding the great efficacy of peptide-based complexes for cellular uptake and mitochondrial targeting [52], as well as with the above reported results of GFP gene expression.

### 3.8. Integrity of Mitochondria

After evidence of ND1 protein production in mitochondria was obtained with the reported delivery systems, we were interested to find out whether the transfection with complexes interfered with the normal mitochondrial function—especially with regard to ATP production. To investigate this issue, measurements of ATP in isolated mitochondria of HeLa cells were performed after transfection with peptide/pND1 complexes, as these complexes offered higher performance as pND1 delivery systems to mitochondria in comparison with the complexes based on PEI–DQA. A luminescent ATP detection kit was used (experimental details are available in the SM). Figure S6 (see the SM) shows the luminescence levels found in the mitochondria of cancer cells after transfection with peptide complexes. Although there was a statistically significant difference in luminescence levels in relation to control cells, peptide complexes such as the ones based on MTS-(KH)_9_ and CpMTP allowed us to maintain the production of high ATP content. This preliminary study demonstrated that, at least, these complexes and the transfection processes they mediated did not greatly interfere with the normal performance of the mitochondria. After transfection, the mitochondria of HeLa cells were able to produce ATP. It seemed that transfection with these peptide/pND1 complexes was an innocuous process for the normal mitochondrial function and, thus, mitochondrial integrity seemed to be preserved. In contrast, the delivery system based on MTS-WRAP1 exhibited a major decrease in ATP levels, in comparison with controls (**** *p* < 0.0001) (Figure S6). The comparison between the MTS-WRAP1/pND1 systems with the other peptide complexes was also statistically significant (**** *p* < 0.0001). 

At this stage, we are therefore aware that in order to deeply study the efficiency of the proposed peptide/pND1 complexes for mitochondrial gene therapy, the design/conception of ND1-mutated disease models is imperative. This would bring a realistic platform to further evaluate the capacity of peptide-based delivery vectors for long-term ND1 transgene expression in the mitochondria. 

## 4. Conclusions

Mitochondrial gene therapy has emerged as a potential therapeutic strategy for mitochondrial diseases originating from mtDNA mutations. To make this therapy feasible and clinically viable, the design and conception of a mitochondria-targeted gene delivery system is crucial. To this end, we report in this work the development of novel polymeric complexes able to load/encapsulate a mitochondrial gene, to target mitochondria, and to promote gene delivery and protein expression in this organelle. It was found that their physicochemical properties and cytotoxic profile can be optimized by varying the N/P ratio and PEI molecular weight parameters. In addition to these PEI-based complexes, CPPs with mitochondrial targeting specificity, deeply studied in a previous work for pND1 encapsulation and delivery [18], were also evaluated for mitochondrial gene delivery and protein expression. It was found that both polymeric and peptide/pND1 complexes promoted efficient transfection, with consequent gene expression and ND1 protein production in the mitochondria. Therefore, both nano-platforms can be further explored in the quest for a suitable gene delivery system to mediate mitochondrial gene therapy. Moreover, a comparison between polymeric and peptide/pND1 complexes revealed that the peptide-based ones—mainly due to their greater ability for pND1 complexation—displayed superior performance in terms of cellular uptake, gene delivery, and protein expression. Collectively, our results bring significant and relevant knowledge, instigating progress towards mitochondrial transfection mediated by pDNA complexes, as a powerful tool to fight against mitochondrial DNA diseases.

## Data Availability

Not applicable.

## Supplementary Materials

The following supporting information can be downloaded at https://www.mdpi.com/article/10.3390/pharmaceutics14040757/s1, Table S1: GPC data of synthesized polymers; Table S2: Average zeta potential, mean size, and PdI values for PEI–DQA (10 kDa or 25 kDa)/TAT/pND1, MTS-CPP/pND1, and CpMTP/pND1 complexes; Figure S1: FTIR spectra of PEI–DQA (10 kDa and 25 kDa) and dequalinium chloride; Figure S2: GPC thermogram of PEI (10 kDa), PEI (10 kDa)–SA, PEI (10 kDa)–DQA, PEI (25 kDa), PEI (25 kDa)–SA, and PEI (25 kDa)–DQA; Figure S3: CMC graph of PEI–DQA (25 kDa) and PEI–DQA (10 kDa); Figure S4: Confocal microscopy study of the transfection of HeLa cells with PEI–DQA (10 kDa)/TAT/pND1 complexes at an N/P ratio of 20:2:1; Figure S5: Confocal microscopy study of the transfection of HeLa cells with PEI–DQA (25 kDa)/TAT/pND1 complexes at an N/P ratio of 20:2:1; Figure S6: Detection of ATP in the mitochondria of HeLa cells after transfection mediated by MTS-WRAP1/pND1, MTS-WRAP5/pND1, MTS-(KH)_9_/pND1, or CpMTP/pND1 complexes at an N/P ratio of 5.
