# Supplementary material for: Peptides vs. Polymers: Searching for the Most Efficient Delivery System for Mitochondrial Gene Therapy
Source: Pharmaceutics. 2022 Mar 31;14(4):757. doi: 10.3390/pharmaceutics14040757 (PMC9026848; doi:10.3390/pharmaceutics14040757)
Supplement: Supplementary file 1 [file pharmaceutics-14-00757-s001.zip › pharmaceutics-1625444-supplementary.pdf]

pharmaceutics

MDPI

Supplementary Material

Peptides vs. Polymers: Searching for the Most Efficient Delivery System for Mitochondrial Gene Therapy

Rúben Faria, Milan Paul, Swati Biswas, Eric Vivès, Prisca Boisguérin, Ângela Sousa and Diana Costa

1. Experimental Section

1.1. Fourier transform infrared spectroscopy.

The functional groups present in the synthesized polymer were characterized by FTIR analysis using an FTIR (Jasco-4200, USA) by the KBr pellet method. The samples were prepared by triturating PEI-DQA (10, 25 KDa) and potassium bromide (KBr) at the weight ratio of 1:99, respectively, to form a pellet. The pellet was placed in the sample holder. FTIR spectra of samples were recorded by scanning pellets over a range of 4000 to 400 cm -1.

1.2. Gel Permeation Chromatography (GPC).

Determination of the molecular weight of synthesized PEI-SA-DQA was carried out using gel permeation chromatography. Ultrahydrogel linear (7.8 mm ID × 300 mm × 6 μ) size exclusion column was used to elute the samples in GPC system (Waters Alliance series). Water was used as a mobile phase with flow rate of 1 ml/min. The standard molecular weight compounds were run before analyzing the sample to plot the calibration curve.

1.3. Determination of Critical Micelles Concentrations (CMC).

CMC of PEI-DQA (10, 25 KDa) was assessed by pyrene incorporation method. Briefly, to glass vials containing 0.5 mg of pyrene, PEI-DQA (10, 25 KDa) solutions were added at various concentrations (3.125-150 μg/mL). The solution was shaken overnight for pyrene dissolution. The solutions were filtered using polycarbonate membranes (0.45 μm). The fluorescence was analyzed using a fluorescence plate reader (Spectramax, microplate reader, Molecular Devices, California, USA) at wavelengths λex 339 and λem 390 nm. CMC was calculated from the inflection point in the fluorescence (I339/I390) vs. log concentration graph.

1.4. Detection of ATP in mitochondria of HeLa cells.

ATP produced in mitochondria of HeLa cells after transfection with peptide/pND1 complexes, at N/P ratio of 5, has been determined by using the Luminescent ATP detection kit from Abcam (ab113849; Abcam, Cambridge, UK), and following instructions provided by the manufacturer. This assay involved the lysis of the cell sample, addition of luciferase enzyme and luciferin, followed by measurement of the emitted light. Briefly, ATP standard was added to standard wells in the same plate containing control (untreated cells) and samples to be analyzed. After transfection with the various complexes, mitochondria of HeLa cells (1 x 10^4) were isolated from cytosol, transferred to 6-well plates (2 mL per well) and then detergent solution was added followed by 5 min incubation, at room temperature, to lyse the cells and stabilize ATP. Then, substrate solution was added, incubated for 5 min at 25 °C and plates were stored in dark for 10 min. After, luminescence was quantified using a luminescence microplate reader (Molecular Devices, Sunnyvale, CA, USA).

2. Results

Table S1. Gel Permeation chromatography data of the polymers.

Name	Mn	Mw	Mp	PDI	Number of molecules attached
PEI 25kDa	12759	24842	19429	1.946	-
PEI 25kDa-SA	22782	26523	29529	1.1640	17.97
PEI 25kDa-SA-DQA	25268	33092	19944	1.3096	12.45
PEI 10kDa	7832	9682	6523	1.3520	-
PEI 10kDa-SA	8875	11542	10256	1.4652	17.03
PEI 10kDa-SA-DQA	9469	17856	11452	1.0652	11.02

Table S2. Average zeta potential, size and PdI for PEI-DQA(10 kDa or 25 kDa)/TAT/pND1, MTS-CPP/pND1 and CpMTP/pND1 complexes.

**PEI 10 kDa**	**Zeta Potential (mV)**	**Size (nm)**	**PdI**
PEI-DQA/TAT/pND1 R10	-5.74 ± 0.11	478± 6	0.54 ± 0.03
PEI-DQA/TAT/pND1 R20	-4.56 ± 0.02	456 ± 3	0.53 ± 0.02
PEI-DQA/TAT/pND1 R50	-4.29 ± 0.36	447 ± 4	0.57 ± 0.04
PEI-DQA/TAT/pND1 R100	-3.97 ± 0.12	388 ± 7	0.61 ± 0.03
PEI-DQA/TAT/pND1 R200	-3.48 ± 0.28	361 ± 1	0.44 ± 0.02
PEI-DQA/TAT/pND1 R500	-2.94 ± 0.12	349 ± 4	0.43 ± 0.02
**PEI 25 kDa**			
PEI-DQA/TAT/pND1 R10	-4.60 ± 0.23	455 ± 6	0.61 ± 0.03
PEI-DQA/TAT/pND1 R20	-3.66 ±0.34	434 ± 4	0.56 ± 0.02
PEI-DQA/TAT/pND1 R50	-2.94 ±0.58	409 ± 7	0.50 ± 0.04
PEI-DQA/TAT/pND1 R100	-2.19 ±0.10	359 ± 4	0.42 ± 0.05
PEI-DQA/TAT/pND1 R200	+3.20 ±0.23	332 ± 5	0.41 ± 0.03
PEI-DQA/TAT/pND1 R500	+5.23 ±0.27	270 ± 6	0.41 ± 0.05
**MTS-WRAP1 [18]**			
MTS-WRAP1/pND1 N/P 1	-1.83 ± 0.90	406 ± 19	0.42 ± 0.06
MTS-WRAP1/pND1 N/P 2	+1.33 ± 0.75	366 ± 14	0.37 ± 0.02
MTS-WRAP1/pND1 N/P 3	+6.50 ± 0.76	276 ± 13	0.23 ± 0.01
MTS-WRAP1/pND1 N/P 5	+11.50 ± 0.76	197 ± 8	0.27 ± 0.02
**MTS-WRAP5 [18]**			
MTS-WRAP5/pND1 N/P 1	-2.17 ± 0.90	399 ± 12	0.49 ± 0.04
MTS-WRAP5/pND1 N/P 2	+7.17 ± 0.90	316 ± 10	0.22 ± 0.03
MTS-WRAP5/pND1 N/P 3	+10.83 ± 1.07	266 ± 8	0.36 ± 0.02
MTS-WRAP5/pND1 N/P 5	+19.33 ± 1.60	175 ± 11	0.30 ± 0.03
**MTS-(KH)9 [18]**			
MTS-(KH)9/pND1 N/P 1	+3.17 ± 0.69	400 ± 14	0.44 ± 0.03
MTS-(KH)9/pND1 N/P 2	+5.67 ± 0.75	366 ± 11	0.38 ± 0.03
MTS-(KH)9/pND1 N/P 3	+8.50 ± 0.50	309 ± 9	0.27 ± 0.04
MTS-(KH)9/pND1 N/P 5	+14.67 ± 0.75	220 ± 9	0.23 ± 0.01
**CpMTP [18]**			
CpMTP/pND1 N/P 1	+2.00 ± 0.58	402± 25	0.56 ± 0.05
CpMTP/pND1 N/P 2	+3.50 ± 0.50	385 ± 14	0.24 ± 0.04

CpMTP/pND1 N/P 3	+5.83 ± 0.69	313 ± 10	0.30 ± 0.02
CpMTP/pND1 N/P 5	+12.67 ± 0.75	236 ± 13	0.23 ± 0.02

Footnotes: Complexes of MTS-CPP/pND1 and CpMTP/pND1 were previously published [18]. The values were calculated with data obtained from three independent measurements (mean ± SD, n = 3).

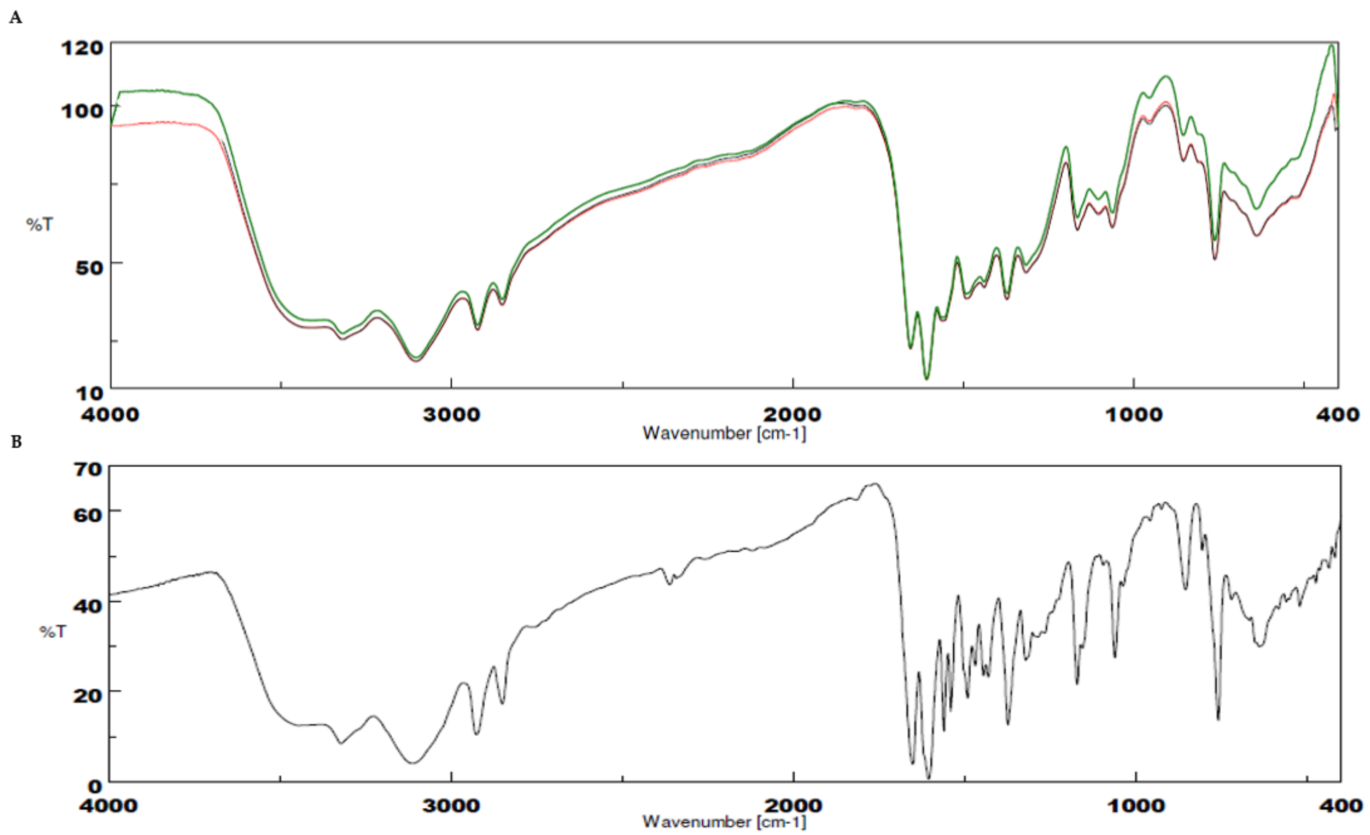

Figure S1. FT-IR spectra of PEI-DQA (10kDa green line and 25kDa red line) (A) and Dequalinium chloride (B).

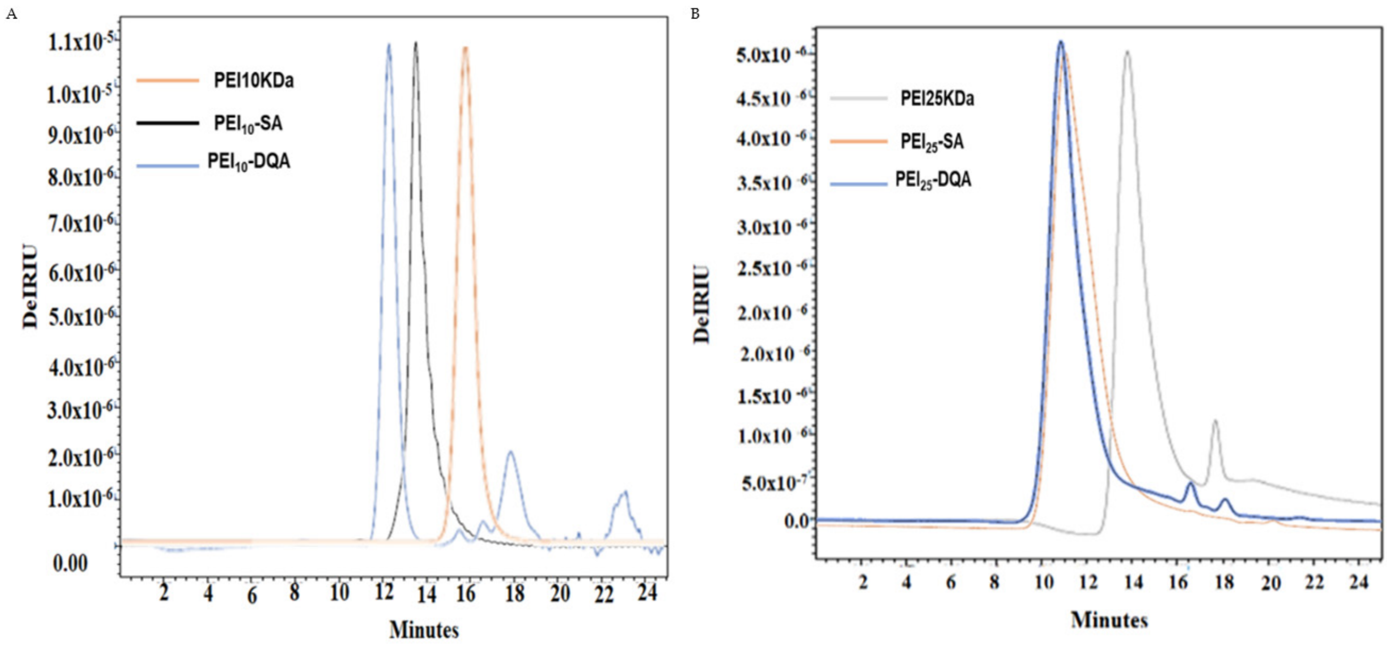

Figure S2. GPC thermogram of PEI 10kDa, PEI 10kDa-SA, PEI 10kDa-DQA (A) and PEI 25kDa, PEI 25kDa-SA, PEI 25kDa-DQA (B).

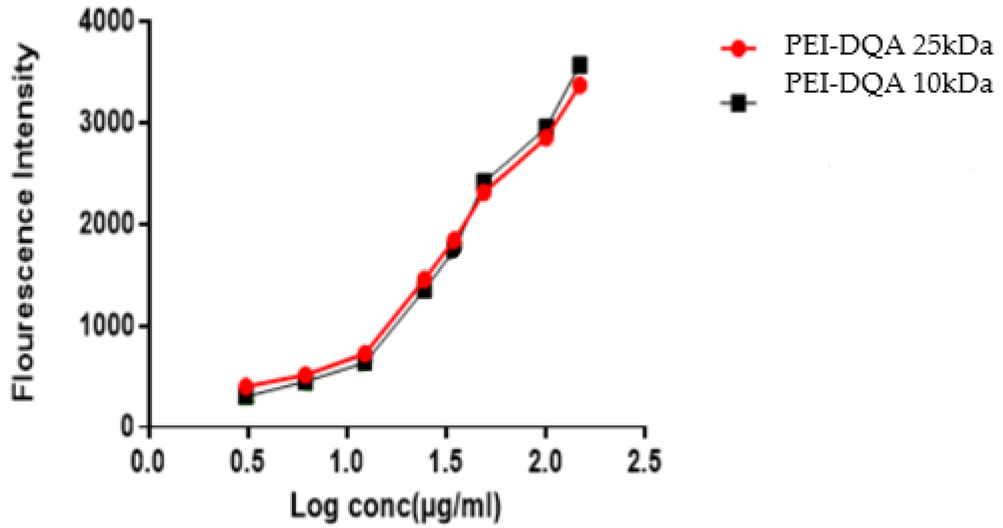

Figure S3. CMC graph of PEI-DQA 25kDa and PEI-DQA 10kDa.

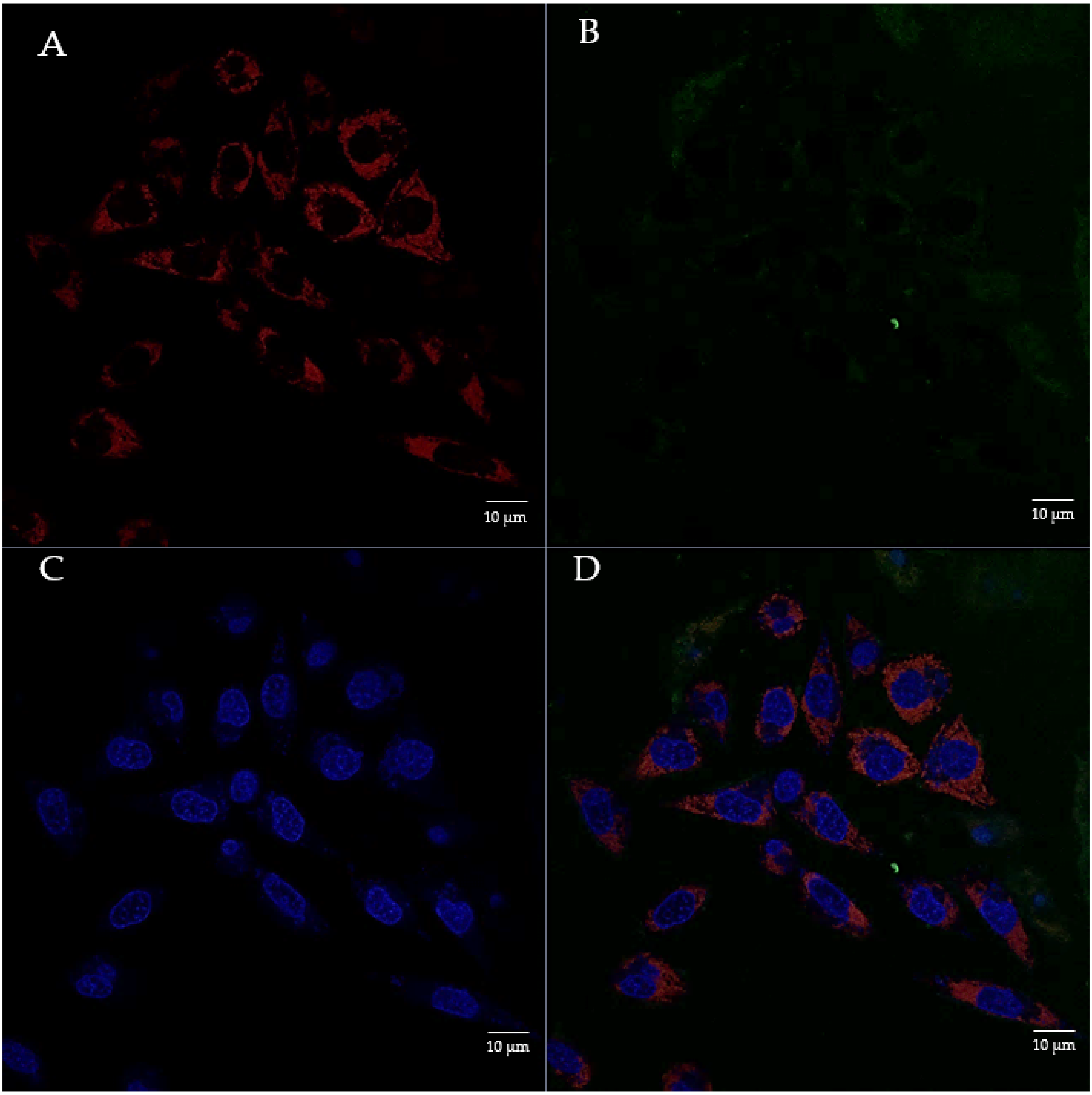

Figure S4. Cellular uptake and intracellular co-localization of PEI-DQA(10kDa)/TAT/pND1 complexes formulated at N/P ratio of 20:2:1. Mitochondria stained red by MitoTracker (A) pND1 green ~~labelled~~ labeled (B), Nucleus marked blue by DAPI (C) and Merged image (D).

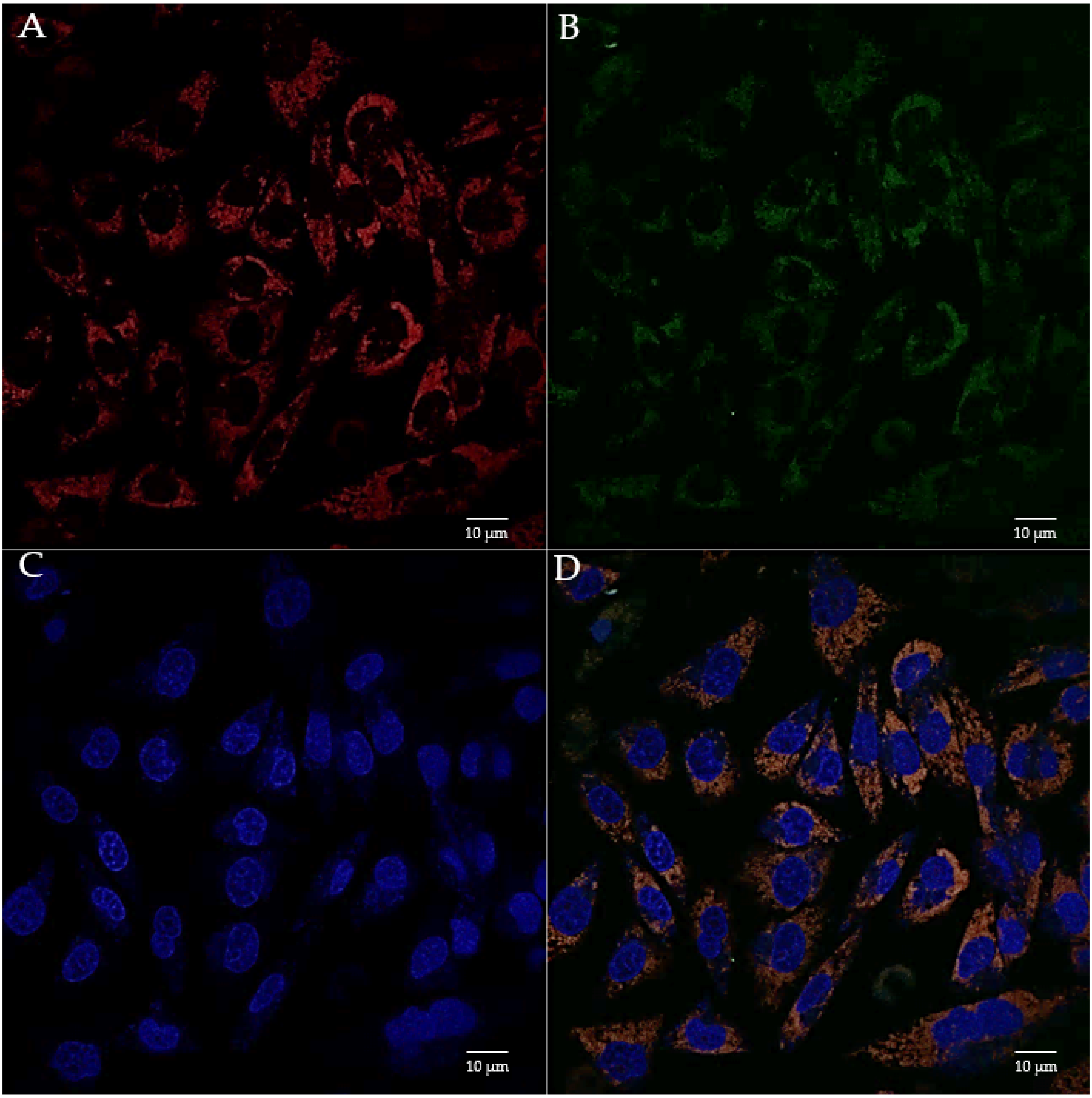

Figure S5. Cellular uptake and intracellular co-localization of PEI-DQA(25kDa)/TAT/pND1 complexes formulated at N/P ratio of 20:2:1. Mitochondria stained red by MitoTracker (A) pND1 green labeled (B), Nucleus marked blue by DAPI (C) and Merged image (D).

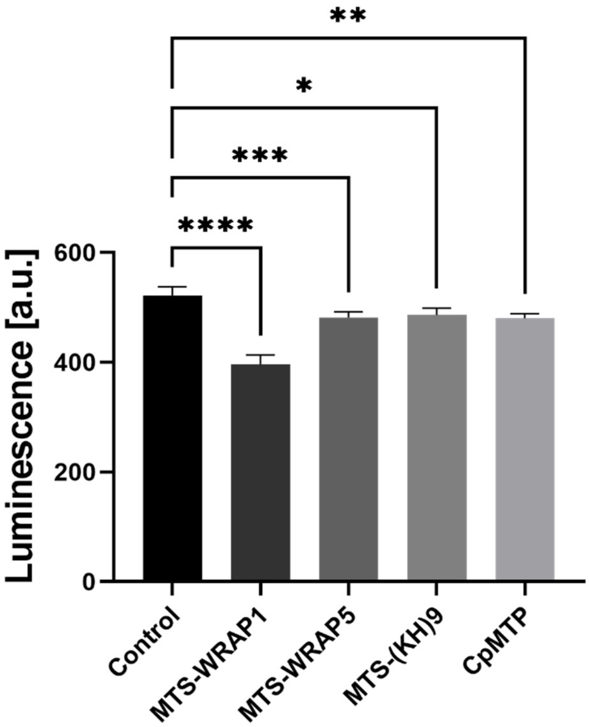

Figure S6. Detection of ATP in mitochondria of HeLa cells after 48 h transfection mediated by MTS-WRAP1/pND1, MTS-WRAP5/pND1, MTS-(KH)9/pND1 or CpMTP/pND1 complexes, all prepared at N/P ratio of 5. Luminescence levels (arbitrary units, a. u.) were determined by using ATP luminescence kit (ab113849). Non-transfected cells were used as control. The values were calculated with the data obtained from three independent measurements (mean ± SD, n = 3) and analyzed by one-way ANOVA followed by Bonferroni test.
